# Causal relationship between gut microbiota and risk of gastroesophageal reflux disease: a genetic correlation and bidirectional Mendelian randomization study

**DOI:** 10.3389/fimmu.2024.1327503

**Published:** 2024-02-21

**Authors:** Kui Wang, Suijian Wang, Yuhua Chen, Xinchen Lu, Danshu Wang, Yao Zhang, Wei Pan, Chunhua Zhou, Duowu Zou

**Affiliations:** ^1^ Department of Gastroenterology, Ruijin Hospital, School of Medicine, Shanghai Jiao Tong University, Shanghai, China; ^2^ Department of Gastroenterology, The First People’s Hospital of Yunnan Province, The Affiliated Hospital of Kunming University of Science and Technology, Kunming, Yunnan, China; ^3^ Department of Endocrinology and Metabolism, First Affiliated Hospital of Stantou University Medical College, Stantou, China; ^4^ The First Clinical Medical College, Lanzhou University, Lanzhou, Gansu, China; ^5^ Cardiology Department, Geriatrics Department, Foshan Women and Children Hospital, Foshan, Guangdong, China

**Keywords:** causal association, gastroesophageal reflux disease, genome-wide association study, comprehensive bidirectional mendelian randomization, gut microbiota

## Abstract

**Background:**

Numerous observational studies have identified a linkage between the gut microbiota and gastroesophageal reflux disease (GERD). However, a clear causative association between the gut microbiota and GERD has yet to be definitively ascertained, given the presence of confounding variables.

**Methods:**

The genome-wide association study (GWAS) pertaining to the microbiome, conducted by the MiBioGen consortium and comprising 18,340 samples from 24 population-based cohorts, served as the exposure dataset. Summary-level data for GERD were obtained from a recent publicly available genome-wide association involving 78 707 GERD cases and 288 734 controls of European descent. The inverse variance-weighted (IVW) method was performed as a primary analysis, the other four methods were used as supporting analyses. Furthermore, sensitivity analyses encompassing Cochran’s Q statistics, MR-Egger intercept, MR-PRESSO global test, and leave-one-out methodology were carried out to identify potential heterogeneity and horizontal pleiotropy. Ultimately, a reverse MR assessment was conducted to investigate the potential for reverse causation.

**Results:**

The IVW method’s findings suggested protective roles against GERD for the *Family Clostridiales Vadin BB60 group* (*P* = 0.027), *Genus Lachnospiraceae UCG004* (P = 0.026), *Genus Methanobrevibacter* (*P* = 0.026), and *Phylum Actinobacteria* (P = 0.019). In contrast, *Class Mollicutes* (*P* = 0.037), *Genus Anaerostipes* (P = 0.049), and *Phylum Tenericutes* (*P* = 0.024) emerged as potential GERD risk factors. In assessing reverse causation with GERD as the exposure and gut microbiota as the outcome, the findings indicate that GERD leads to dysbiosis in 13 distinct gut microbiota classes. The MR results’ reliability was confirmed by thorough assessments of heterogeneity and pleiotropy.

**Conclusions:**

For the first time, the MR analysis indicates a genetic link between gut microbiota abundance changes and GERD risk. This not only substantiates the potential of intestinal microecological therapy for GERD, but also establishes a basis for advanced research into the role of intestinal microbiota in the etiology of GERD.

## Introduction

Gastro-esophageal reflux disease (GERD) prevalently affects both adult and pediatric cohorts (1, 2). The worldwide incidence of GERD is rising substantially (3). The predominant phenotype of this condition is non-erosive reflux disease (NERD) (4, 5). NERD is typified by the hallmark symptoms of GERD, yet devoid of esophageal erosion. GERD syndromes encompass typical reflux symptoms, characterized by heartburn and regurgitation, potentially accompanied by belching, water brash, or nausea. Additionally, manifestations may include chest pain resembling angina and extra-oesophageal symptoms like chronic cough and laryngitis (6–8). Moreover, persistent gastroesophageal reflux may result in the transformation of the distal esophagus’s stratified squamous epithelium to columnar epithelium, precipitating the onset of Barrett’s esophagus (BE) (9). BE, characterized by the presence of metaplastic columnar mucosa in the distal esophagus, heightens the risk of cancer. This condition is uniquely identified as the antecedent to esophageal adenocarcinoma, a malignancy whose prevalence has surged notably in the preceding decades (10–13). Hence, numerous researchers aim to devise prevention strategies for esophageal adenocarcinoma by investigating the pathogenesis of GERD and Barrett’s esophagus (14, 15). The human gastrointestinal tract is host to a complex and varied microbiota, which holds a pivotal function in health and pathophysiology. This includes processes such as the digestion and assimilation of nutrients, production of vital vitamins like B and K, *in vivo* degradation of molecules, orchestration of innate and adaptive immune reactions, and preservation of the intestinal barrier’s integrity (16–18).

In recent years, numerous studies have elucidated the correlation between the onset and progression of various intestinal diseases and the intestinal flora (19). Consequently, scholars have redirected their attention to the study of esophageal microbiota, aiming to elucidate the pathogenesis, early detection, and therapeutic approaches for esophageal disorders. It has been noted that the esophageal microflora composition varies markedly between GERD-affected and normal esophagus. A preliminary research conducted by Yang in 2009 identified a potential association between modifications in the distal esophageal microbiome and disorders related to reflux. Bacterial populations from 34 patients were analyzed using 16S rRNA gene sequencing following biopsies of the distal esophagus. Based on gene analysis outcomes, the authors delineated the human esophageal microbiome into two categories. Type I esophageal microbiome corresponded more closely with the normal esophagus, whereas Type II was more associated with the pathological esophagus (20). Studies indicate a heightened colonization of Gram-negative organisms, particularly Campylobacters, in the esophageal mucosa of GERD patients compared to healthy cohorts (21). Dysregulation of the mycobiota has been implicated in the onset of visceral hypersensitivity, a condition closely associated with intractable symptoms of GERD (22). These observations prompt consideration of potential dysbiosis involvement in the pathogenesis of GERD ailments. In observational research, the relationship between the gut microbiota and GERD is susceptible to confounding variables, including dietary habits, environmental factors, age, and lifestyle. These confounders complicate the process of establishing a direct causal link between gut microbiota and GERD. Utilizing the Mendelian randomization (MR) approach allows for the inference of causative associations between exposures and subsequent outcomes (23, 24). This methodology employs genes as instrumental variables (IVs), which, due to their reliance on the random assortment of genetic variation at conception, are less prone to confounding influences (25). In the present research, we executed a two-sample MR analysis to assess the putative causal relationship between the gut microbiota and GERD. Through this endeavor, we aspire to elucidate novel perspectives on the potential involvement of the gut microbiome in the pathogenesis of GERD and discern potential pathways for preventative and therapeutic strategies. To our knowledge, this is the first time that Mendelian randomization has been used to study the pathogenic impact of the gut microbiome on the pathogenesis of GERD.

## Materials and methods

### Study design

**Figure 1:**
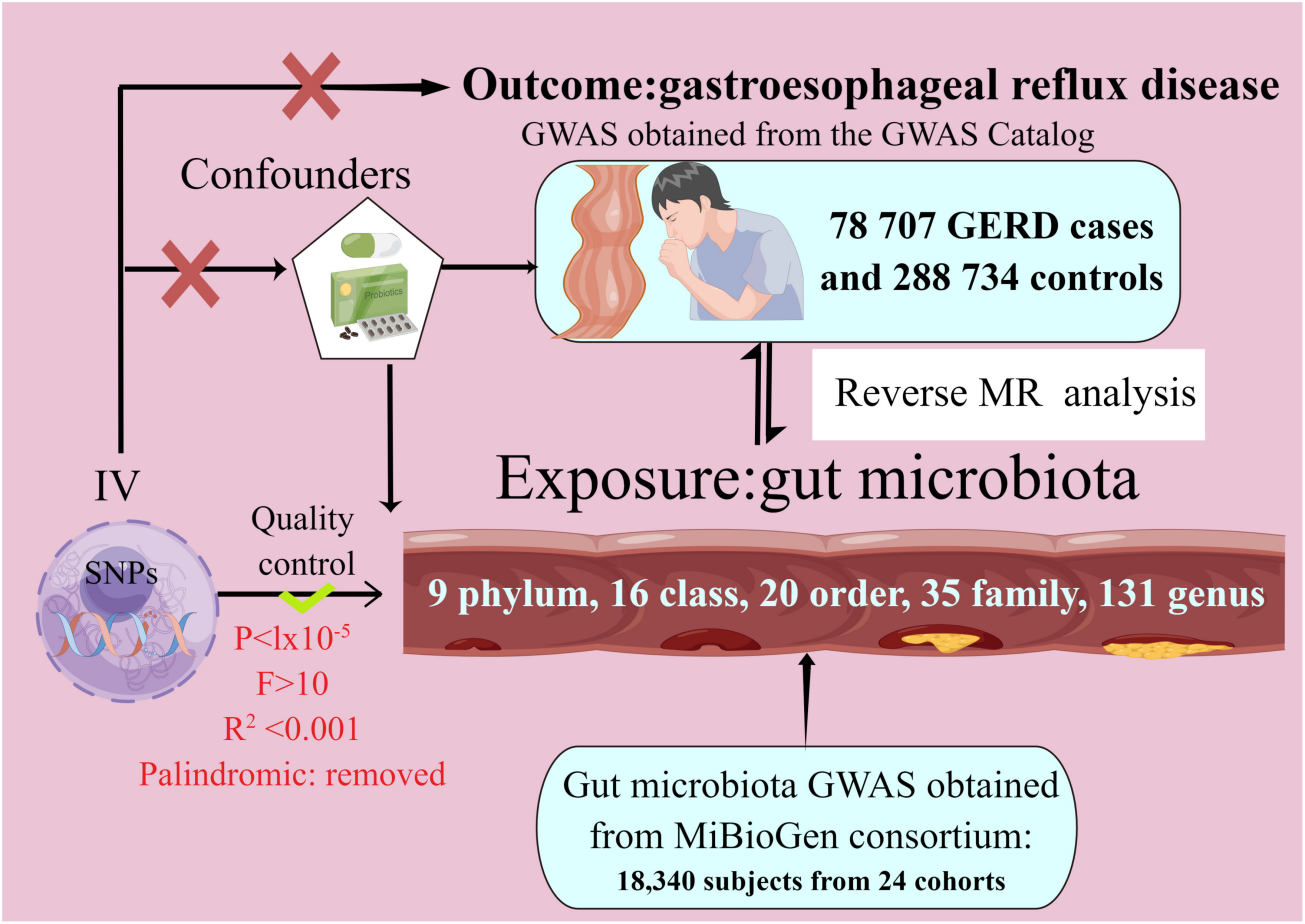
The study design of the present Mendelian randomization study of the associations of the gut microbiota and GERD risk.

In our study, we performed two-sample MR analyses with gut microbiota as the exposure and GERD as the outcome. To investigate the causal relationship between intestinal microflora and GERD, we utilized a bi-sample MR approach, drawing on data from the MiBioGen consortium (N = 18,340) and recent GWAS (78 707 GERD cases and 288 734 controls) findings. **Figure 1** depicts the MR study flowchart detailing the relationship between GM taxa and GERD. For reliable results, the MR study adhered to these three assumptions (1). They are significantly associated with the exposure (2); They don’t influence the confounders linking exposure and outcome; and (3) They don’t impact the outcome via alternative pathways (26). The current MR study was executed and chronicled in accordance with the STROBE-MR guidelines, established to enhance the reporting caliber of observational epidemiological investigations (27–29).

### Data sources

Gut microbiota and GERD data were sourced from GWAS datasets. The intestinal microbiome information came from the MiBioGen consortium’s GWAS analysis, which included 18,340 individuals spanning 24 whole-genome genotype cohorts and 16S fecal microbiome data (30). We gathered summary-level data on SNP-GERD associations from the recent publication’s GWAS results. This analysis encompassed 78,707 GERD cases and 288,734 controls of European ancestry (31). GERD is characterized by abnormal esophageal acid exposure leading to GERD symptoms and/or mucosal injury due to gastro-oesophageal reflux.

### Selection of SNPs

We conducted quality control procedures to select appropriate instrumental variants (IVs) (32–35). SNPs associated with each microbiota unit, meeting the locus-wide significance threshold (*P*< 1.0 × 10^−5^), were designated as potential IVs. The linkage disequilibrium (LD) assessment among these SNPs is as follows (36–38): LD denotes the non-random co-occurrence of alleles at distinct loci. It is evaluated via two metrics, r^2^ and kb. An r^2^ value spans from 0 to 1, with lower values signifying a heightened level of complete linkage equilibrium between two SNPs, suggesting a stochastic arrangement of these SNPs. An appropriate LD window size and r^2^ threshold are selected to guarantee independence, given the profound impact of linkage disequilibrium. SNPs were clumped for independence using the European 1000 Genomes Project reference panel with criteria r^2^ < 0.001 and clump distance > 10,000 kb. SNPs exhibiting a Minor Allele Frequency (MAF) of 0.01 or lower were systematically excluded from the analysis. We excluded both redundant and palindromic SNPs from our analysis. To ensure a robust association between instrumental variables (IVs) and exposure measures, the F-statistic of each SNP was employed to evaluate the strength of correlation, mitigating potential biases from weak IVs. IVs were considered devoid of bias if the F-statistic exceeded 10. To minimize the likelihood of SNPs being associated with potential confounders or risk determinants (e.g., coronary heart disease, Idiopathic pulmonary fibrosis), the Phenoscanner tool was utilized to meticulously assess and exclude such correlations.

### MR analysis and quality assessment

We derived the primary MR estimates using the inverse-variance weighted (IVW) method. We also assessed the robustness of these IVW findings by contrasting them with results from other MR techniques, such as MR-Egger, weighted median, simple mode, and weighted mode estimation. The analyses conducted encompassed evaluations of heterogeneity, an assessment of horizontal pleiotropy, and a systematic leave-one-out examination. For the assessment of heterogeneity, the Cochrane’s Q test was employed, with a *P*-value of less than 0.05 being considered indicative of significant heterogeneity. The Mendelian Randomization Pleiotropy Residual Sum and Outlier (MR-PRESSO) approach, in conjunction with the MR-Egger method, were utilized to scrutinize horizontal pleiotropy. A *P*-value of less than 0.05 was deemed indicative of the presence of horizontal pleiotropy. we performed a leave-one-out analysis to evaluate the results’ sensitivity, wherein each SNP was sequentially excluded to determine if the estimates were influenced by outliers or bias. We determined the statistical power for MR analysis by utilizing the mRnd web application, accessible at https://shiny.cnsgenomics.com/mRnd/ (39). In particular, for the purpose of refining our outcomes in the context of multiple hypotheses, we employed both the Bonferroni correction method and the Hochberg’s False Discovery Rate (FDR) approach. The criterion for deeming results statistically significant was established on the basis of a P-value less than 0.05, adjusted by dividing it by the effective count of unique bacterial taxa present at the respective taxonomic level, a value hereinafter referred to as ‘n’, An association was deemed statistically significant in instances where the p-value, after undergoing Bonferroni correction, was found to be below the threshold of 0.05. Conversely, the presence of a p-value lesser than 0.05, which nonetheless corresponded to a Bonferroni-corrected p-value exceeding 0.05, was interpreted as indicative of suggestive, rather than conclusive, evidence of an association.

### Reverse MR analysis

To investigate the putative causal association between GERD and distinct bacterial genera, a reverse MR analysis was undertaken. In this context, GERD was posited as the exposure variable, while the gut microbiota composition functioned as the outcome variable. SNPs associated with GERD were utilized as instrumental variables in this analytical framework. SNPs that exhibited a statistically significant association with GERD were selected as instrumental variables, adhering to a significance threshold of *P* < 5 × 10^−8^.

### Ethical approval

written informed consents were meticulously secured from all participating individuals. Concurrently, these investigations were granted the requisite endorsements from the pertinent ethical oversight bodies (30).

## Results

In the current research, preliminary endeavors were undertaken to procure high-quality IVs through stringent quality assurance measures. Subsequently, these IVs were employed in a MR analysis to evaluate the presumptive causal association between 196 gut microbiota taxa and GERD. In each retained SNP, the F-statistic surpassed a threshold of 10, as delineated in the **Supplementary Tables S1**, **S2**. The statistical efficacy of MR analysis was greater than 70%.This indicates a robust statistical strength in the association between the IV and its respective bacterial taxa. For all MR results, we conducted comprehensive sensitivity analyses to assess both heterogeneity, as denoted by Cochran’s Q statistic, and potential pleiotropic influences, as appraised via MR-Egger regression and the MR-PRESSO approach. The P-values were subjected to a more stringent Bonferroni correction, and all results were greater than 0.05.

### Causal effect of gut microbiota on GERD

**Figure 2:**
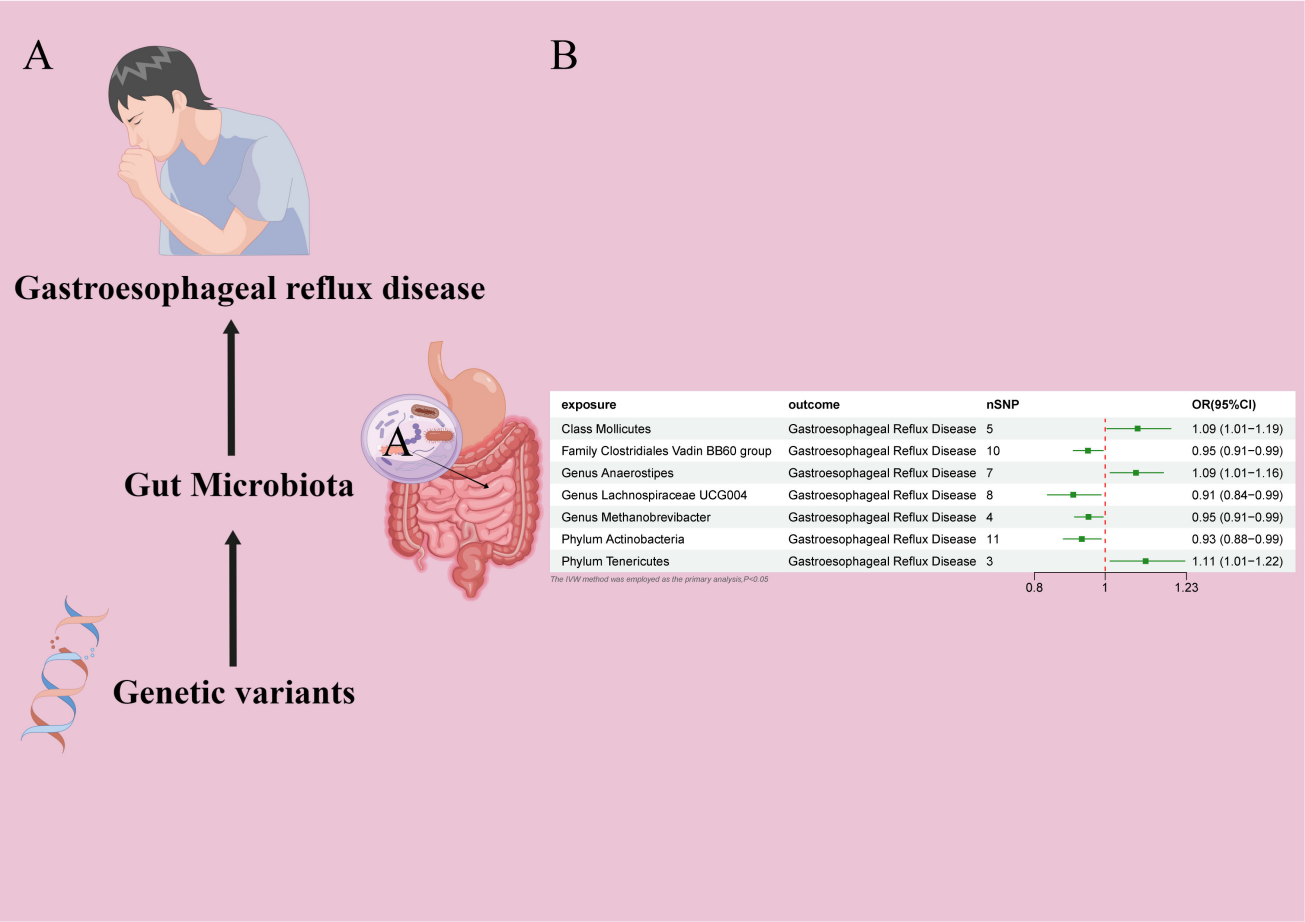
**(A)** Causal effect of gut microbiota with GERD Schematic representation of the MR analysis results **(B)** Forest plot of the MR analysis results.

**Figure 3:**
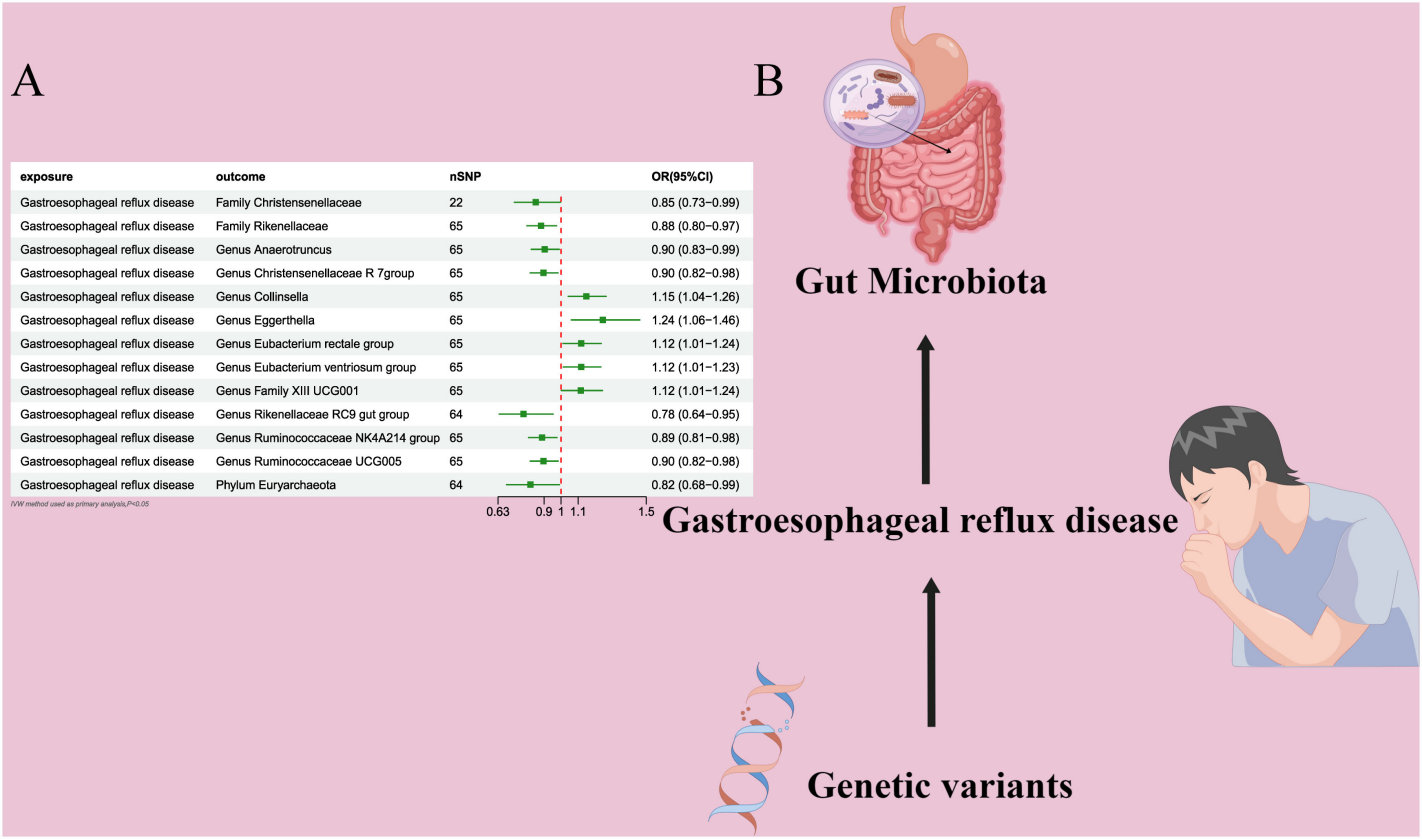
**(A)** Forest plot of the MR analysis results. **(B)** Forest plot of the MR analysis results Causal effect of GERD with gut microbiota Schematic representation of the Reverse MR analysis results. OR odds ratio, CI confidence interval, IVW inverse variance weighted method, Significant threshold was set at *P*-value <0.05 for the Inverse Variance Weighted method (IVW).

**Figure 4:**
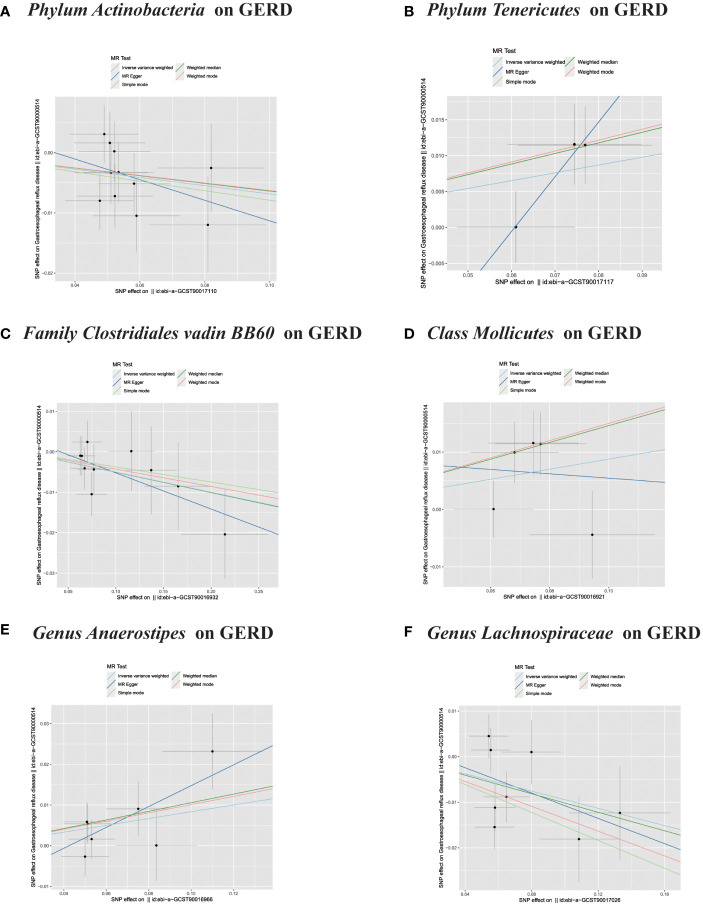
**(A–F)** Scatter plots of significant causality of the GM and GERD.

**Figure 5:**
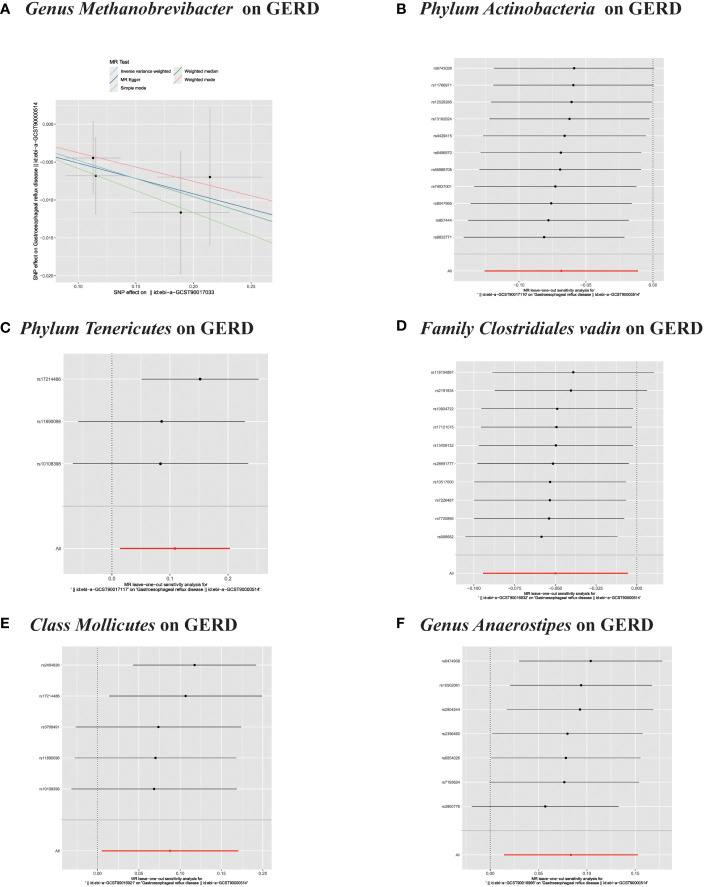
**(A)** Scatter plots of significant causality of the GM and GERD. **(B–F)** Leave-one-out analysis for the impact of individual SNPs on the association between GM and GERD risk.

**Figure 6:**
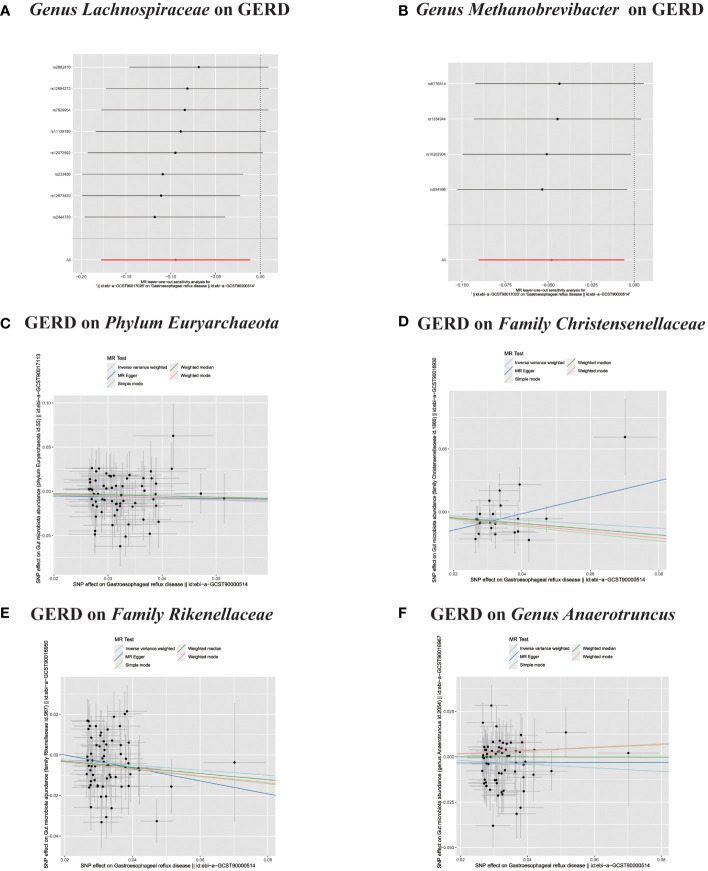
**(A, B)** Leave-one-out analysis for the impact of individual SNPs on the association between GM and GERD risk. **(C–F)** In reverse MR analysis, The scatter plots for association between GERD and gut microbiota.

In the MR study on gut microbiota, employing microbiota-linked SNPs as instrumental variables, the primary IVW analysis identified seven taxa with a probable causal association to GERD onset. Through the application of the IVW analytical approach, the following associations with GERD susceptibility were discerned: The *Family Clostridiales Vadin BB60 group* (OR 0.95, 95% CI 0.91–0.99, P = 0.027), *Genus Lachnospiraceae UCG004* (OR 0.91, 95% CI 0.84–0.99, P = 0.026), *Genus Methanobrevibacter* (OR 0.95, 95% CI 0.91–0.99, P = 0.026), and *Phylum Actinobacteria* (OR 0.93, 95% CI 0.88–0.99, P = 0.019) manifested an inverse correlation with GERD vulnerability. In contrast, the *Class Mollicutes* (OR=1.09, 95% CI:1.01–1.19, P=0.037); *Genus Anaerostipes* (OR=1.09, 95% CI:1.01–1.16, P=0.017) and *Phylum Tenericutes* (OR=1.11, 95% CI:1.01–1.22, P=0.024) demonstrated association with the risk of GERD. (**Figures 2**, **3**) The *P*-values obtained from both the Cochran Q test and the MR-Egger intercept test surpassed the 0.05 threshold. This provides robust evidence indicating an absence of heterogeneity and pleiotropy in the research (**Table 1**; **Supplementary Table 2**, **Figures 2**; **4**–**6**).

**Table 1:** Summary results of MR (Target Gut microbiome on GERD).

Taxa	Exposure	Outcome	Nsnp	Methods	Beta	SE	OR (95%CI)	*P* value	Heterogeneity		Horizontal pleiotrop	
									Cochran’s Q	P value	Egger intercept P	MR-PRESSO P
Phylum	Actinobacteria	GERD	11	Inverse variance weighted	-0.068	0.029	0.93 (0.88-0.99)	0.019	6.835	0.740	0.579	0.78
Phylum	Tenericutes	GERD	3	Inverse variance weighted	0.108	0.048	1.11 (1.01-1.22)	0.024	2.468	0.291	0.364	NA
Family	Clostridiales vadin BB60 group	GERD	10	Inverse variance weighted	-0.049	0.022	0.95 (0.91-0.99)	0.027	4.406	0.882	0.490	0.85
Class	Mollicutes	GERD	5	Inverse variance weighted	0.087	0.042	1.09 (1.01-1.19)	0.037	6.032	0.196	0.745	0.27
Genus	Anaerostipes	GERD	7	Inverse variance weighted	0.083	0.035	1.09 (1.01-1.16)	0.017	5.506	0.480	0.246	0.49
Genus	Lachnospiraceae UCG004	GERD	8	Inverse variance weighted	-0.09	0.042	0.91 (0.84-0.99)	0.026	13.72	0.056	0.789	0.14
Genus	Methanobrevibacter	GERD	4	Inverse variance weighted	-0.047	0.021	0.95 (0.91-0.99)	0.026	0.333	0.953	0.931	0.95

### Causal effect of GERD on gut microbiota

**Figure 7:**
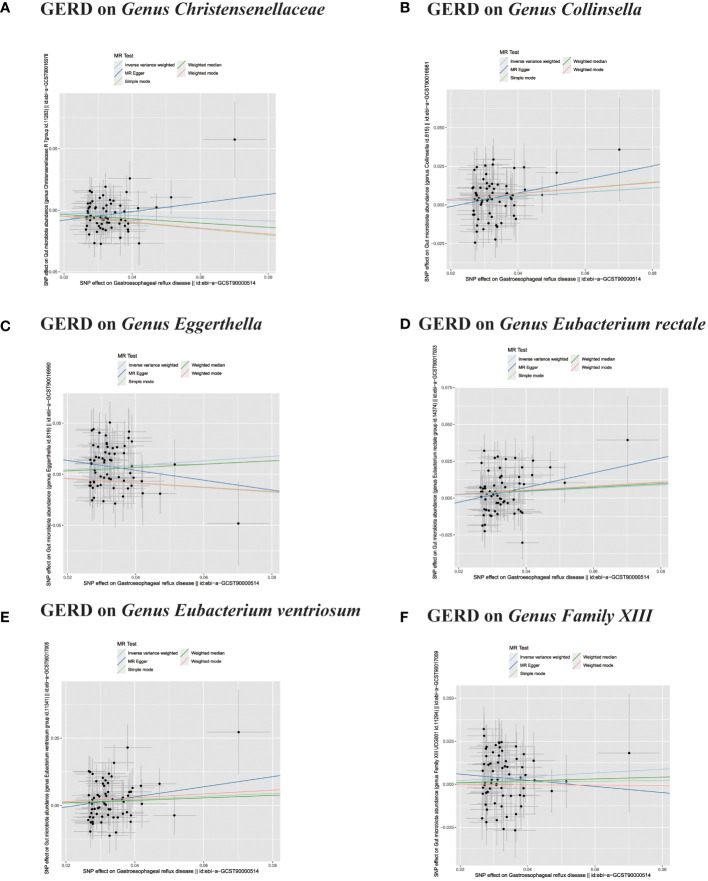
**(A–F)** In reverse MR analysis, The scatter plots for association between GERD and gut microbiota.

**Figure 8:**
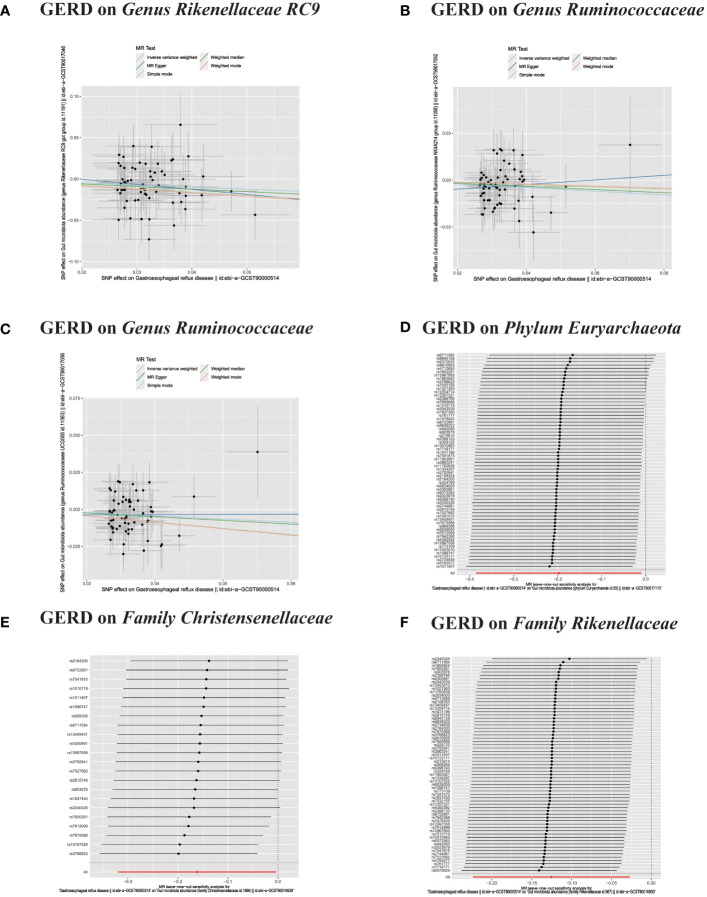
**(A–C)** In reverse MR analysis, The scatter plots for association between GERD and gut microbiota. **(D–F)** In reverse MR analysis, Plots for "leave-one-out" analysis for causal effect of GERD on gut microbiota risk;.

**Figure 9:**
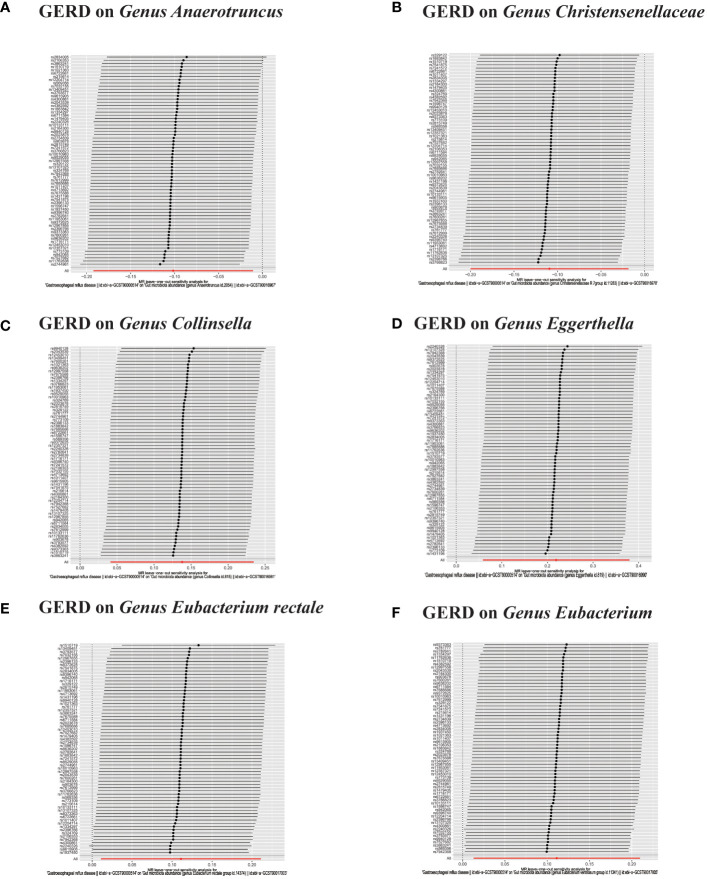
**(A–F)** In reverse MR analysis, Plots for “leave-one-out” analysis for causal effect of GERD on gut microbiota risk.

**Figure 10:**
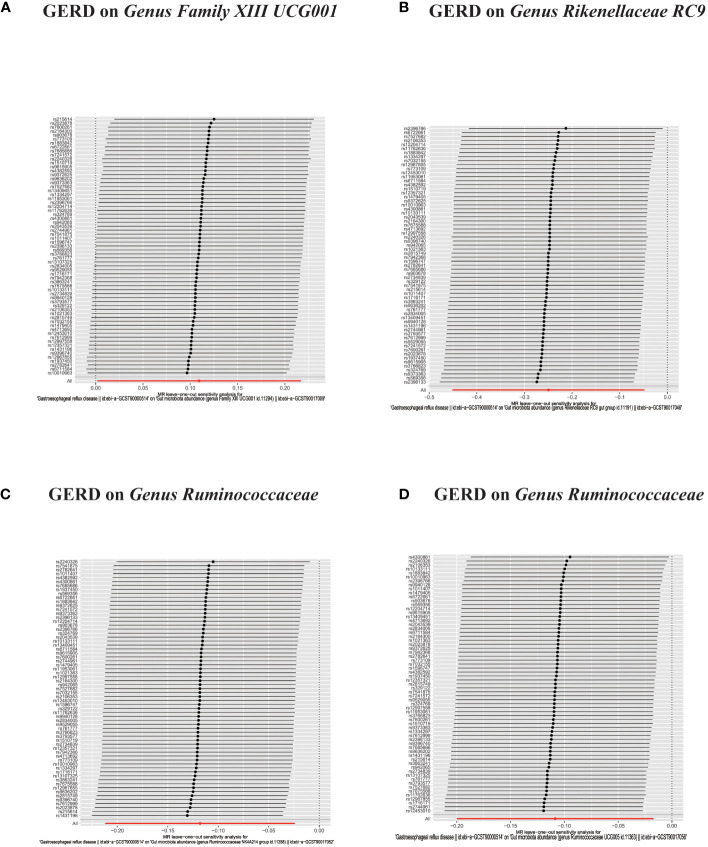
**(A–D)** In reverse MR analysis, Plots for "leave-one-out" analysis for causal effect of GERD on gut microbiota risk.

In the bidirectional MR analysis, we explored the potential causal association between GERD and gut microbiota. Employing GERD as the exposure and gut microbiota as the outcome, we evaluated potential reverse causation implications. Following the MR analysis, GERD exhibited a causal influence on one Phylum, two Families, and ten Genera. Utilizing the IVW approach, several associations with the onset of GERD were identified. Specifically, a down-regulation was observed in the *Family Christensenellaceae* (OR=0.85, 95% CI:0.73–0.99, P=0.045), *Family Rikenellaceae* (OR=0.88, 95% CI:0.80–0.97, P=0.012), *Genus Anaerotruncus* (OR=0.90, 95% CI:0.83–0.99, P=0.028), *Genus Christensenellaceae R 7 group*(OR=0.90, 95% CI:0.83–0.99, P=0.018), *Genus Rikenellaceae RC9 gut group* (OR=0.78, 95% CI:0.64–0.95, P=0.015), *Genus Ruminococcaceae NK4A214 group* (OR=0.89, 95% CI:0.81–0.98, P=0.013), *Genus Ruminococcaceae UCG005* (OR=0.90, 95% CI:0.82–0.98, P=0.019), and *Phylum Euryarchaeota* (OR=0.82, 95% CI:0.68–0.99, P=0.039). Conversely, an up-regulation post GERD onset was documented for *Genus Collinsella* (OR=1.15, 95% CI:1.04–1.26, P=0.005), *Genus Eggerthella* (OR=1.24, 95% CI:1.06–1.46, P=0.007), *Genus Eubacterium rectale group* (OR=1.12, 95% CI:1.01–1.24, P=0.029), *Genus Eubacterium ventriosum group* (OR=1.12, 95% CI:1.01–1.23, P=0.026), and *Genus Family XIII UCG001* (OR=1.12, 95% CI:1.01–1.24, P=0.046) (**Figures 4**, **5**). Within the IVs, neither weak instrument bias nor significant heterogeneity metrics were identified. Further, the MR-PRESSO evaluation indicated no discernible outliers. The data’s robustness was further affirmed by the leave-one-out analysis (**Table 2**; **Figures 3**, **6**–**10**).

**Table 2:** Summary results of bidirectional MR (GERD on target Gut microbiome).

Exposure	Taxa	Outcome	Nsnp	Methods	Beta	SE	OR (95%CI)	*P* value	Heterogeneity		Horizontal pleiotrop	
									Cochran’s Q	P value	Egger intercept P	MR-PRESSO P
GERD	Phylum	Euryarchaeota	64	Inverse variance weighted	-0.197	0.095	0.82 (0.68-0.99)	0.039	49.131	0.899	0.859	0.904
GERD	Family	Christensenellaceae	22	Inverse variance weighted	-0.161	0.080	0.85 (0.73-0.99)	0.045	22.259	0.384	0.052	0.398
GERD	Family	Rikenellaceae	65	Inverse variance weighted	-0.125	0.050	0.88 (0.80-0.97	0.012	81.301	0.071	0.527	0.072
GERD	Genus	Anaerotruncus	65	Inverse variance weighted	-0.101	0.046	0.90 (0.83-0.99	0.028	64.943	0.443	0.708	0.438
GERD	Genus	Christensenellaceae R 7group	65	Inverse variance weighted	-0.109	0.046	0.90 (0.82-0.98)	0.018	56.898	0.723	0.110	0.712
GERD	Genus	Collinsella	65	Inverse variance weighted	0.137	0.049	1.15 (1.04-1.26)	0.005	58.047	0.685	0.325	0.71
GERD	Genus	Eggerthella	65	Inverse variance weighted	0.219	0.082	1.24 (1.06-1.46)	0.007	57.356	0.708	0.147	0.706
GERD	Genus	Eubacterium rectale group	65	Inverse variance weighted	0.111	0.051	1.12 (1.01-1.24)	0.029	82.493	0.059	0.216	0.078
GERD	Genus	Eubacterium ventriosum group	65	Inverse variance weighted	0.111	0.050	1.12 (1.01-1.23)	0.026	70.367	0.273	0.391	0.28
GERD	Genus	Family XIII UCG001	65	Inverse variance weighted	0.109	0.054	1.12 (1.00-1.24)	0.046	70.735	0.262	0.395	0.28
GERD	Genus	Rikenellaceae RC9 gut group	64	Inverse variance weighted	-0.249	0.102	0.78 (0.64-0.95)	0.015	53.945	0.784	0.594	0.768
GERD	Genus	Ruminococcaceae NK4A214 group	65	Inverse variance weighted	-0.118	0.047	0.89 (0.81-0.98)	0.013	50.213	0.895	0.366	0.884
GERD	Genus	Ruminococcaceae UCG005	65	Inverse variance weighted	-0.108	0.046	0.90 (0.82-0.98)	0.019	60.194	0.611	0.689	0.6

## Discussion

To our knowledge, this is the first MR study to assess the causal relationship between the gut microbiome and susceptibility to gastroesophageal reflux disease. Using GWAS summary data, we confirmed an association between GERD and the gut microbiome. Our research findings are consistent with extant academic literature, revealing a bidirectional relationship between GERD and the gut microbiome. We identified specific risk factors, including the *Class Mollicutes, Genus Anaerostipes and Phylum Tenericutes*. In contrast, protective factors, such as the *Family Clostridiales Vadin BB60 group,Genus Lachnospiraceae UCG004, Genus Methanobrevibacter and Phylum Actinobacteria*, were observed to be linked with GERD within the gut microbiome. The emergence of GERD manifested alterations in the gut microbiome composition. Following the MR analysis, GERD exhibited a causal influence on one Phylum, two Families, and ten Genera. Furthermore, the Phylum Actinobacteria, Family Clostridiales Vadin, and Genus Methanobrevibacter have been identified as contributors to the biosynthesis of Short-chain fatty acids (SCFAs). SCFAs emerge from the bacterial fermentation of indigestible dietary fibers within the gastrointestinal tract. The primary constituents of SCFAs are acetate, propionate, and butyrate. These acids not only serve as a principal energy source for colonocytes but also play a pivotal role in the dual-directional regulation of colonic motility, the preservation of intestinal homeostasis, and the enhancement of the integrity of the intestinal barrier (40–42). The human gastrointestinal epithelium is inhabited by a myriad of microbial entities that are instrumental in multiple physiological processes. An imbalance within this microbial composition, termed intestinal dysbiosis, has been intricately linked to the etiology of numerous human pathologies. Innate lymphoid cells (ILCs), encompassing NK cells, ILC1s, ILC2s, ILC3s, and LTi cells, represent a subset of the innate immune system. Predominantly localized within the body’s mucosal tissues, these cells have lately been the subject of significant academic scrutiny (43). Research has demonstrated a correlation between the presence of Clostridiales and a spectrum of esophageal pathologies, including esophagitis and BE. This association is hypothesized to influence the inflammatory processes of the esophageal mucosa and contribute to the development of intestinal metaplasia (44–46).

Recently, numerous research endeavors have delved into the association between gut microbiota and GERD. Ning L et al. documented a diminished prevalence of the *phylum Actinobacteria* in GERD patients, a result that is congruent with the findings of this study (47, 48). research indicated a substantial elevation in the levels of Proteobacteria and Bacteroidetes in pediatric subjects suffering from GERD. Concurrently, there was a notable decrease in the concentrations of Firmicutes and Actinobacteria (49). A Japanese research endeavor employed a distinctive method using quantitative 16S rRNA gene PCR to ascertain total bacterial quantities. The findings suggest that the relative proportions of taxa, including *Proteobacteria, Firmicutes, Bacteroidetes, Fusobacteria, and Actinobacteria*, hold greater relevance to esophageal disorders than the absolute bacterial counts (47).

Our study initially demonstrated that the Family *Clostridiales Vadin BB60 group*, *Genus Methanobrevibacter*, and *Genus Lachnospiraceae UCG004* function as protective agents against GERD. These results underscore the putative roles of distinct gut microbiome entities in the pathogenesis of GERD, further accentuating the imperative for comprehensive studies to elucidate the foundational mechanisms and identify prospective therapeutic avenues. The hypothesis posits bacterial biofilm’s role in GERD etiology (21). A recent investigation identified differential microbiota in NERD patients relative to control individuals and those with esophageal adenocarcinoma (EAC). Researchers employed 16S rRNA sequencing and mass spectrometry-based proteomics to profile the esophageal microbiota and the host mucosal proteome, respectively. An aggregate of 70 individuals spanning four patient categories (NERD, reflux esophagitis, Barrett’s esophagus, and EAC) along with a control group were examined. The findings revealed a singular microbiota configuration in NERD, divergent from the control and other cohorts (50). Proton pump inhibitors (PPI) remain a foundational component in the therapeutic approach to reflux disease. Modifications in the esophageal microbiome due to the diminished gastric acidity induced by PPI have been investigated in multiple research endeavors, illustrating their consequential impact on microbial community configurations (51–61). The gut microbiota comprises an extensive array of microorganisms residing in the human gastrointestinal tract, facilitating various physiological and biochemical processes for the host (62). Alterations in the composition of esophageal microbiota can be attributed to environmental influences. A diet rich in fats has been strongly correlated with localized mucosal inflammatory modifications in murine representations (63). The postulated mechanism for this advantage is the decelerated fermentation, resulting in enhanced luminal accessibility in contrast to conventional fiber-laden products. The preliminary investigation demonstrated notable beneficial impacts of sugarcane flour on alleviating GERD symptoms, necessitating a more expansive randomized controlled trial (64).Probiotics introduce bacterial strains via dietary supplementation, aiming to optimize the gut microbiota composition towards a more favorable equilibrium. Evaluations of probiotics encompassing Lactobacilli spp. and Bifidobacteria spp. have shown efficacy in alleviating GERD manifestations (65–68). This research seeks to determine a causal link between particular gut microbiota and GERD through MR analysis. Comprehending the relationship between gut microbial dysbiosis and the onset of GERD, as well as pinpointing the specific gut microbiota associated with GERD, can facilitate the proactive identification of individuals at elevated risk. This understanding permits the prompt initiation of targeted preventative measures and the tailoring of clinical interventions, which can mitigate symptoms such as regurgitation and heartburn. Furthermore, such approaches can enhance patients’ overall well-being and curtail economic burden.

Our study possesses key strengths. Firstly, MR represents an analytical methodology employing genetic variants as IVs to elucidate the causal relationship between exposure and outcome. The MR framework mitigates unobserved confounders and counteracts reverse causation, which are prevalent in observational research. Second, we employed the most extensive GWAS pertaining to the gut microbiota currently available, though its sample size remains notably constrained (n = 14,306). Prospective GWAS investigations concerning the gut microbiota should endeavor to augment the sample size to conventional GWAS benchmarks (n > 100,000) to enhance statistical power and minimize potential inaccuracies. Our research, admittedly, possesses certain limitations. First, A segmented analysis considering overarching determinants like age and gender was not feasible owing to the constraints inherent in the GWAS summary data. Second, we refrained from adjusting for multiple testing, as stringent corrections for multiple comparisons might overlook strains that have a causal association with GERD. Thirdly, the summary-level data from GWAS predominantly originate from European cohorts, constraining the universal applicability of our results.

In conclusion, while we have postulated a causal link between gut microbiota and GERD at the genetic dimension, the underlying biological pathways warrant further investigation. Our findings may serve as a foundational framework for delving into the mechanisms of specific gut microbiomes in individuals with GERD. In future clinical endeavors, it may be feasible to gauge the prevalence of gut microbiota in fecal samples as a prognostic tool for assessing GERD risk. Additionally, modulating the gut microbiota could serve as a preventive and therapeutic strategy for GERD.

## Conclusion

This research identified certain microbial taxa as either protective or risk determinants for GERD. Such findings may offer valuable biomarkers for diagnostic purposes and potential therapeutic intervention points for GERD. Subsequent research endeavors ought to corroborate these results in human subjects and delve deeper into elucidating the underlying mechanisms.

## Data availability statement

The original contributions presented in the study are included in the article/**Supplementary Material**. Further inquiries can be directed to the corresponding authors.

## Ethics statement

The studies involving humans were approved by written informed consents were meticulously secured from all participating individuals. Concurrently, these investigations were granted the requisite endorsements from the pertinent ethical oversight bodies. The studies were conducted in accordance with the local legislation and institutional requirements. Written informed consent for participation in this study was provided by the participants’ legal guardians/next of kin. Written informed consent was obtained from the individual(s) for the publication of any potentially identifiable images or data included in this article.

## Author contributions

KW: Conceptualization, Data curation, Investigation, Methodology, Software, Supervision, Validation, Visualization, Writing – original draft. SW: Conceptualization, Data curation, Investigation, Methodology, Software, Supervision, Validation, Writing – original draft. YC: Conceptualization, Formal analysis, Investigation, Software, Writing – original draft, Writing – review & editing, Visualization. XL: Conceptualization, Data curation, Investigation, Methodology, Software, Supervision, Validation, Writing – original draft. DW: Conceptualization, Data curation, Investigation, Software, Supervision, Writing – original draft. YZ: Conceptualization, Data curation, Formal analysis, Investigation, Project administration, Software, Supervision, Writing – review & editing. WP: Project administration, Supervision, Data curation, Methodology, Writing – original draft, Writing – review & editing. CZ: Conceptualization, Writing – original draft, Investigation, Data curation, Supervision. DZ: Conceptualization, Data curation, Formal analysis, Funding acquisition, Investigation, Methodology, Project administration, Resources, Supervision, Validation, Writing – review & editing.
